# From the Editor: The Journey to Open Access

**Published:** 2015

**Authors:** Robert C. Groom

**Figure 1:**
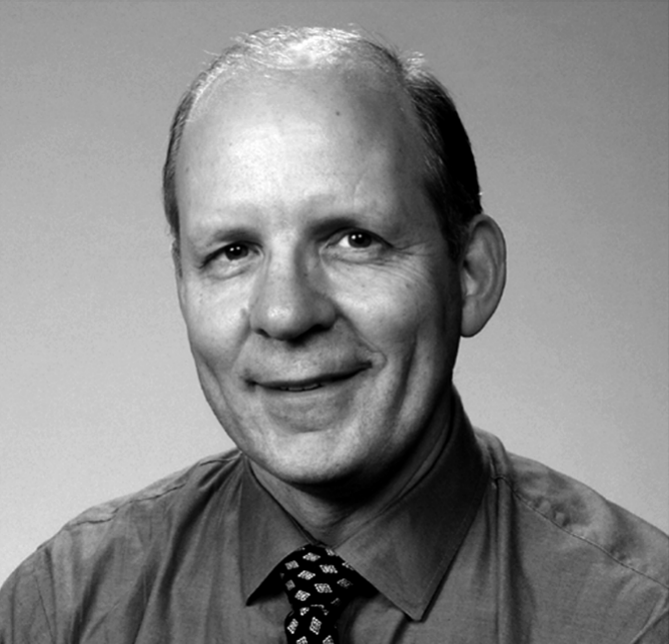
Robert C. Groom

The American Society of ExtraCorporeal Technology (AmSECT) was founded in 1964 with the belief that members of the then-new allied health field could best serve their profession by sharing their thoughts and experiences. The mission of AmSECT, since its charter, has remained the same, “… *to foster improved patient care and safety by providing for the continuing education and professional needs of the extracorporeal circulation technology community*” (1).

*The Journal of ExtraCorporeal Technology* has been one of the principle avenues used by AmSECT in pursuit of its mission through the publication and dissemination of scholarly work since 1967.

A milestone for the Journal was when it became indexed in MEDLINE through PubMed and the Cumulative Index of Nursing and Allied Health Literature in 1990. Since then nearly 1,000 scholarly articles have been indexed and abstracted in the National Library of Medicine’s PubMed database.

Over the last several years AmSECT has strived to form partnerships with other perfusion societies to more broadly disseminate new knowledge and to provide tangible benefits to their members. In 2013, the Australian and New Zealand College of Perfusion (ANZCP) partnered with AmSECT where by *The Journal of ExtraCorporeal Technology* became the Official Journal of the ANZCP. ANZCP members now receive electronic access to the journal, including all archived volumes going back to 1974. This partnership has fostered improved patient care and safety by expanding and disseminating the published knowledge related to extracorporeal technology.

Three years ago, the Journal Editorial Board began deliberate discussion about what could be done to further share our content and it became clear that “open access” was a highly effective avenue to share more. According to Peter Suber, Director of the Harvard Office for Scholarly Communication, open access is compatible with copyright, peer review, revenue (even profit), print, preservation, prestige, quality, career advancement, indexing, and other features associated with conventional scholarly literature (2). Furthermore, open access makes articles more visible, discoverable, retrievable, and useful to the broadest possible audience.

Opening access to the Journal’s content to the world was the right thing to do, and we determined that PubMed Central® (PMC) was the best vehicle to accomplish this objective. PMC is a free archive of biomedical and life sciences journal literature at the U.S. National Institutes of Health’s National Library of Medicine. Participating journals continue to hold copyright of the content, but grant free distribution rights to digital copies of articles from PMC. *The Journal of ExtraCorporeal Technology*’s content, beginning from December 2011, is now archived in PMC. Content published in *The Journal of ExtraCorporeal Technology* is now available free to the world six months after it is first published in the Journal.

Congratulations to the Journal’s Editorial Board for their vision to pursue this avenue to further disseminate new knowledge, and to the AmSECT Board of Directors for approval of this important bold step, an important stride forward in the mission, “*providing for the continuing education and professional needs of the extracorporeal circulation technology community*.”
